# Transforming Plastic Waste into Value: A Review of Management Strategies and Innovative Applications in Sustainable Construction

**DOI:** 10.3390/polym17070881

**Published:** 2025-03-26

**Authors:** Bourhaneddine Haba, Souad Djellali, Yasmine Abdelouahed, Soufiane Boudjelida, Flora Faleschini, Mauro Carraro

**Affiliations:** 1 Department of Sciences, Teacher Education College of Setif (Ecole Normale Supérieure Messaoud Zeghar—Sétif), El Eulma 19600, Setif, Algeria; 2 Laboratory of Non-Metallic Materials, Institute of Optics and Precision Mechanics, University Setif 1 Ferhat Abbas, Setif 19000, Setif, Algeria; 3 Department of Chemistry, Faculty of Sciences, University Setif 1 Ferhat Abbas, Setif 19000, Setif, Algeria; 4 Laboratory of Physical-Chemistry of High Polymers, Faculty of Technology, University Setif 1 Ferhat Abbas, Setif 19000, Setif, Algeria; 5 Department of Industrial Engineering, University of Padova, Via Marzolo 9, 35131 Padova, Italy; 6 Department of Civil, Environmental and Architectural Engineering, University of Padova, Via Marzolo 9, 35131 Padova, Italy; 7 Department of Chemical Sciences, University of Padova, Via Marzolo 1, 35131 Padova, Italy; 8 Institute on Membrane Technology (ITM-CNR), UoS of Padova, Via Marzolo 1, 35131 Padova, Italy

**Keywords:** plastic waste, recycle, management, filler, binder, construction

## Abstract

The world is facing the issue of managing a huge amount of plastic waste. To prevent uncontrolled and unproductive disposal, various valorization strategies have been developed. Recycling plastic waste into valuable composites for construction offers a promising pathway toward sustainable waste management. Given that the construction industry is a major consumer of energy and natural resources, it presents a key opportunity for integrating recycled materials. This review examines diverse strategies and applications for plastic waste recycling, with a particular focus on sustainable construction solutions, while also evaluating the advantages and limitations of this approach. Within this context, recycled plastic waste can be used as a filler to replace non-renewable natural resources. Studies have shown that incorporating plastic waste as a filler improves diverse properties of composites, including thermal and sound insulation. In particular, thermoset plastic waste exhibits desirable characteristics such as rigidity, heat and chemical resistance, strength and durability, making it suitable as a filler for non-structural applications. Alternatively, melting recycled plastic waste can produce binder materials that combine with other inorganic materials to form building and construction composites. Using melted thermoplastic waste as a binder enhances ductility, reduces water absorption, and improves overall durability. Additionally, the hot-pressing technique has been shown to be more effective in addressing poor bonding issues commonly encountered with conventional methods.

## 1. Introduction

Urbanization and industrialization have driven significant global socio-economic progress but at the cost of excessive natural resource consumption and environmental degradation. Managing non-renewable resources remains particularly difficult, as their extraction leads to continuous depletion. Addressing this issue requires a strategic and efficient approach to resource utilization, in order to ensure long-term sustainability.

In the construction sector, innovative solutions should aim to mitigate resource depletion. For example, waste from date palm wood, such as fibers, can be used to reinforce concrete, enhancing its hygrothermal performance and reducing reliance on traditional materials [1,2,3].

Plastics, among the most widely used materials worldwide, are valued for their affordability, lightweight nature, versatility, and ease of molding. However, the vast amount of plastic waste generated annually poses significant environmental and socio-economic challenges. Landfilling, the predominant disposal method, is increasingly unsustainable due to high financial costs and long-term inefficiency in managing the growing waste burden [4,5]. Beyond the limited availability of landfill space, plastic waste poses serious environmental risks. Harmful substances can leach into the soil, contaminating nearby land and groundwater [6]. Additionally, plastic degrades into microplastics (MPs) and nanoplastics (NPs), which infiltrate ecosystems and food chains, posing growing threats to human health and biodiversity [7,8]. Improper disposal further exacerbates these issues, as plastics clog waterways, block drains, and accumulate in urban areas, leading to flooding and pollution [9,10]. Stagnant plastic waste also creates breeding grounds for mosquitoes and other pests, increasing the spread of diseases. Furthermore, plastics release toxic chemicals into the environment, disrupting ecosystems and endangering wildlife [10]. These multifaceted challenges emphasize the urgent need for more effective waste management and recycling strategies.

Prioritizing sustainable practices is critical to mitigating the negative impacts of plastic waste on both the environment and human well-being. Recycling plastic waste to convert it into useful materials is a more attractive strategy, as it not only reduces the need for new resources but also enhances the economic value of the waste [11].

In this context, the present paper explores the recycling of thermoset and thermoplastic plastic waste, focusing on their use as fillers or binders in innovative composites for sustainable construction. On the other hand, while recycling plastic waste into construction materials presents significant opportunities, it is crucial to acknowledge the associated environmental and health challenges [12]. Specifically, pollutants such as heavy metals and volatile organic compounds (VOCs) may be released during the manufacturing of the composites, posing occupational health risks if proper safety measures are not implemented. However, safer additives and methods—currently proposed and under active research—show promising potential for neutralizing or removing harmful plastic substances, thereby minimizing their impact on both human health and the environment [13].

## 2. Classification of Plastic Waste

Polymers are exceptionally versatile materials, typically categorized into thermoplastics, elastomers and thermosets [14,15]. Thermoplastics account for approximately 80% of all consumed plastics, primarily used in packaging and textile fiber production [16], making them the most common type of plastic waste. Thermoplastics consist of polymer chains made of carbon atoms, which can be repeatedly molded and reshaped when exposed to heat [16]. These chains are held together by relatively weak intermolecular forces such as van der Waals forces, dipole–dipole interactions, or hydrogen bonds. When heated, these forces are disrupted, allowing the polymer chains to flow, creating a viscoelastic state. Upon cooling, thermoplastics solidify through processes like vitrification or crystallization (for semi-crystalline polymers) [17]. Compared to materials like silicon or glass, thermoplastics are more cost-effective due to lower raw material costs and efficient manufacturing processes [18]. Their combination of performance, affordability, and design flexibility has made them indispensable across numerous industries [19].

In contrast, thermosets are three-dimensional, cross-linked structures that do not melt or soften when heated [16]. Upon heating, thermosetting plastics undergo irreversible chemical reactions, locking in their final shape once solidified [14,20]. This distinct behavior significantly influences their recyclability and treatment options. Thermosets are typically known for their high hardness and rigidity, offering exceptional heat resistance. Unlike thermoplastics, they exhibit minimal creep or warping under elevated temperatures. Due to their remarkable strength and stiffness, thermosets are often employed in structural applications where they must endure substantial loads and harsh conditions [21].

Thermosets find widespread applications that require durability over extended periods, encompassing composites utilized within the aeronautics and automotive industries, wind turbine blades, and various structural components [22]. Thermoset composites’ market size was valued at USD 46.21 billion in 2024 and is projected to reach USD 122.48 billion by 2031, growing at a CAGR of 7.93% during the forecast period 2024–2031, thus being more relevant than the corresponding global thermoplastic composites’ market size [23].

**Figure 1:**
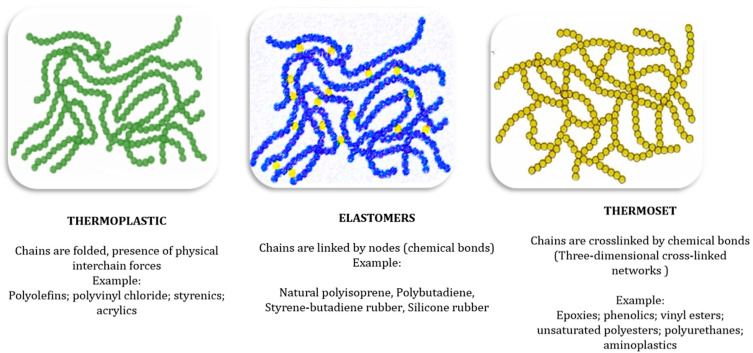
Schematic structure and some properties of thermoplastics, elastomers, and thermosets: thermoplastics exhibit only weak intermolecular forces between polymer chains, while thermosets possess additional cross-linking [16,21].

Figure 1 illustrates the schematics and some properties of thermoplastics and thermosets, with a comparative analysis including elastomers.

As defined by the European Commission (Commission Decision 97/129/EC) [24], seven types of plastics are commonly recycled and identified with codes, as indicated in Table 1, based on their degree of recyclability.

Sogancioglu et al. [27] reported that HDPE, LDPE, PP, PS, and PET constitute 57% of plastic waste, with 43% of this ending up in landfills.

## 3. Environmental Impacts of Plastic Waste

**Figure 2:**
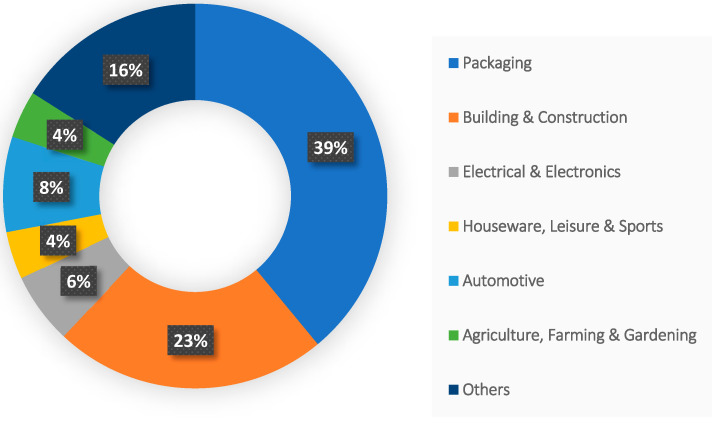
End-use markets for plastics by application (2022, in the EU27 + 3) [26].

The increase in plastic manufacturing during the last seven decades highlights our dependence on this material. The widespread use of plastic is especially prevalent in key sectors such as agriculture, construction, electronics, furniture, consumer products, medical devices, packaging, and transportation [20]. As shown in Figure 2, packaging and construction consistently account for the largest share of plastic consumption.

The rapid growth of the plastics industry is closely connected to the petrochemical sector and the oil industry [25]. In 2020, global production of petroleum-based plastics surged to 367 million tons, up from 1.7 million tons in 1950 [28,29], reflecting its widespread adoption due to versatility and affordability. However, this reliance carries severe environmental consequence [30]. Plastic production depletes finite fossil fuel resources, its molecular structure readily absorbs and transport persistent organic pollutants (POPs), and its durability results in slow degradation and long-term environmental accumulation. Consequently, mismanaged plastic waste has emerged as a significant global pollutant.

**Figure 3:**
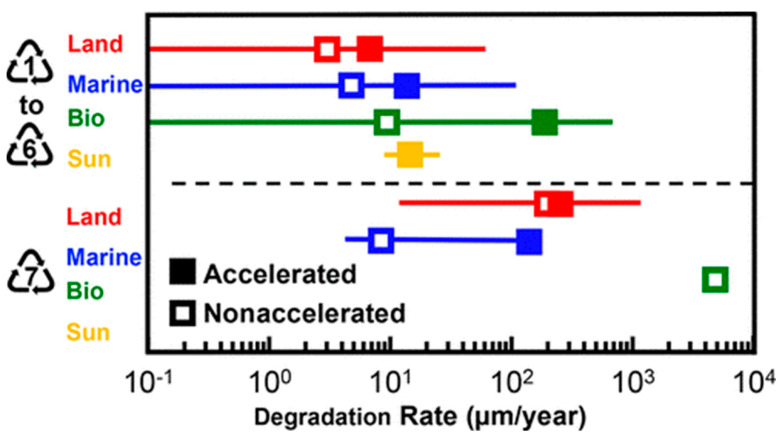
Duration of degradation of some plastic items [31].

As shown in Figure 3, plastics have a lifespan ranging from years to millennia, depending on their properties and environmental conditions [31]. Factors like chemical composition, molecular weight, surface-area-to-volume ratio, and functional groups (which determine hydrophilicity or hydrophobicity) influence degradation rates [32]. Due to their resistance to decomposition, plastics dominate landfills and dumps [33]. According to the United Nations (UNEP), of the 9.2 billion tons of plastic produced between 1950 and 2017, approximately 7 billion tons became waste, accumulating in landfills or dumps [34]. Unlike metals or wood, plastics resist natural decomposition, breaking into smaller fragments over time (microplastics) [28,29]. This degradation is influenced by environmental conditions and the physicochemical properties of the polymer [32].

A significant consequence of plastic waste proliferation is its growing presence in the oceans, threatening marine ecosystems that sustain over 3.5 billion people. Nearly 700 marine species are already endangered by plastic pollution, and this number is expected to rise as more plastic enters the ocean. A 2022 UNESCO report estimates that 8 to 10 million metric tons of plastic are dumped into the ocean annually, contributing to the staggering 50 to 75 trillion pieces of plastic and microplastics currently contaminating marine environments. These plastics either break down into microscopic fragments, infiltrating ecosystems, or form vast floating garbage patches, posing severe ecological threats [35].

Plastics particle are categorized by size [36]: macroplastics (>2.5 cm), mesoplastics (5 mm–2.5 cm), microplastics (MPs, <5 mm), and nanoplastics (NPs, <100 nm) [33]. MPs and NPs, in particular, have become emerging contaminants in water, food, and ecosystems, releasing harmful additives like stabilizers and plasticizers as they degrade [14,37]. If current trends continue, the Earth could accumulate 34 billion tons of plastic by 2050 [38]. Projections estimate that by then, 9 billion metric tons of plastic waste will be recycled, 12 billion incinerated, and another 12 billion either landfilled or released into the environment [39].

In 2019, plastic contributed to the generation of 1.8 billion metric tons of greenhouse gas emissions annually throughout its lifecycle (i.e., 3.4% of global GHG emissions) [40]. Under a business-as-usual scenario, the plastics lifecycle could be responsible for as much as 19% of global greenhouse emissions by 2040 [41]. If plastic were classified as a nation, it would rank as the fifth-largest emitter of greenhouse gases globally [42].

## 4. Socioeconomic Impacts of Plastic Waste

**Figure 4:**
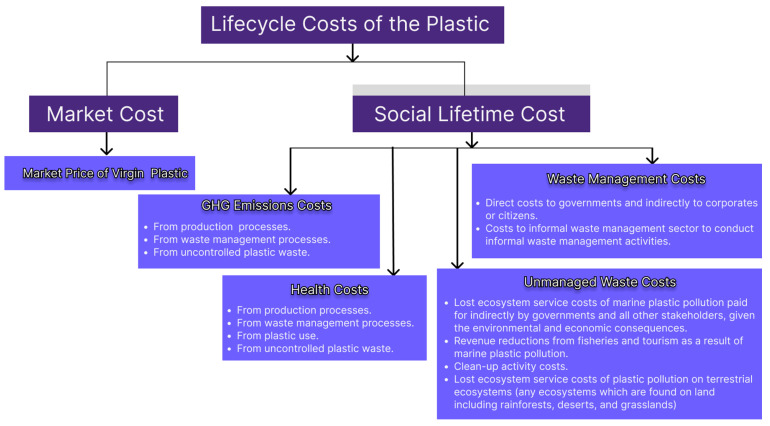
Costs imposed by the plastic lifecycle [42].

Plastic waste exerts significant negative impacts throughout this extended duration, leading to substantial economic and societal costs. A report by Dalberg, commissioned by WWF [42], identifies these costs as encompassing the market value of plastic, expenses associated with greenhouse gas (GHG) emissions, health-related expenditures, waste management costs, and the financial implications of improperly managed waste (Figure 4). Some of these costs, such as those related to waste management, are classified as direct economic costs, while others are indirect, placing considerable pressure on governments and societies by adversely affecting environmental integrity and public health [42].

**Figure 5:**
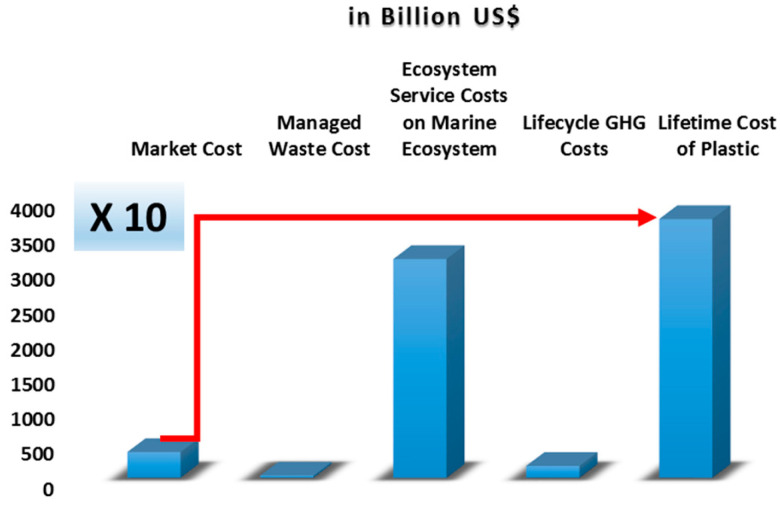
Lifetime cost of plastic produced in 2019 [42].

According to a previous report, in 2019, the estimated minimum cost associated with the plastic produced throughout its lifetime is approximately USD 3716 billion. Moreover, the total lifetime cost associated with plastic produced in 2019 is ten times higher than its market price (Figure 5). The report shows that, without intervention, this cost is projected to double for the plastic generated by the year 2040.

Throughout its lifecycle, plastic is a major contributor to greenhouse gas emissions, with these emissions amounting a cost of more than USD 171 billion in 2019; this figure represents over one-third of the global expenditure on energy transitions in the year 2020 [42].

It can be inferred that plastic pollution incurs costs across all aspects of life [43]:

- The management of plastic waste entails significant financial expenditures, with annual costs surpassing USD 32 billion. These expenses encompass activities such as collection, sorting, disposal, and recycling processes.
- Clean-up efforts to remove waste incur significant costs for governments, non-governmental organizations, and concerned citizens, reaching as high as USD 15 billion annually.
- The substantial economic impact of marine plastic pollution includes a significant reduction in gross domestic product (GDP), reaching an estimated USD 7 billion in 2018 alone. This is primarily attributed to revenue losses in tourism, fishing, and aquaculture.
- Marginalized communities disproportionately bear the environmental and health burdens of the plastic lifecycle. Incineration plants and refineries are often sited in these areas, exposing residents to significant health and economic risks. Informal waste pickers face severe health risks throughout the plastic waste processing cycle. Additionally, climate change—exacerbated by plastic production and degradation—disproportionately affects disadvantaged communities, further deepening social and environmental inequities.

## 5. Plastic Waste Management Strategy

According to the United Nations Environment Program (UNEP), between 1950 and 2017, only a small fraction of plastic waste, between 9% and 15%, was managed, while the majority, around 80%, ended up in landfills or the environment [34]. This highlights the immense potential for applying circular economy principles to plastic waste management [10].

The 4Rs waste management strategy prioritizes actions based on their environmental benefits, in the following order: reduce, reuse, recycle (materials), and recover (energy), with landfilling being the least desirable option [25]. Reduction focuses on minimizing waste generation through careful consumption, while reuse involves repurposing items or components that retain functional value. Recycling is the process of transforming waste materials into new resources [44].

Plastic recycling specifically refers to convertingplastic waste into useful products, often in forms that differ significantly from the original [45]. Recycling plastic offers significant benefits, including reducing GHG emissions [46]; indeed, enhancing recycling efforts could lead to net-negative GHG emissions by decreasing the demand for virgin raw materials and avoiding the emissions associated with their production. Additionally, plastic recycling helps mitigate environmental impacts and reduces the volume of plastic waste directed to landfills [47]. Plastic recycling is broadly categorized into four distinct types, which will also be discussed in more detail in the next paragraph [25]:

1. Primary: Mechanical reprocessing into a product with equivalent properties (often referred to as closed-loop recycling). This technique is applicable to single-type plastics that are relatively clean or uncontaminated; for example, PET can be recycled from used bottles to create new bottles [48].
2. Secondary: Mechanical reprocessing into products requiring lower properties (referred to as downgrading). Recycling and contamination with other polymers can lower the molecular weight of plastics, leading to a decline in mechanical properties. Furthermore, the reprocessing itself can cause thermo-mechanical degradation in recycled plastics. An example of secondary recycling is the production of flooring tiles from mixed polyolefins [48].
3. Tertiary: Recovery of chemical constituents (it is referred to as either chemical recycling or feedstock recycling, and it occurs when the polymer is de-polymerized to its starting constituents). Pyrolysis is another example of this approach [48].
4. Quaternary: Recovery of energy (described as energy from waste or valorization). This technique significantly reduces waste volume and involves energy recovery through the incineration of waste plastics. It is typically used when the waste is highly contaminated and unsuitable for conventional recycling methods [48].

Substituting virgin plastic with recycled plastic presents a significant challenge, primarily influenced by two key factors: the purity of the recovered plastic feedstock and the performance requirements of the desired plastic product [25]. It is important to understand that plastic-recycling methods are diverse, and their effectiveness varies significantly depending on the polymer type and design [25]: for example, recycling is significantly simpler and more economical for rigid containers made from a single type of polymer than for those with multiple layers or components. Thermoplastics, like HDPE, LDPE, PET, and PP (Table 1), can be effectively identified and sorted from waste streams, enabling easy and economical mechanical recycling processes [25]. This makes these plastics the most frequently recycled types [49]. In contrast, thermosetting polymers, like unsaturated polyester or epoxy resin, cannot be readily recycled through traditional melting and re-shaping processes; however, they can be repurposed as filler materials after being ground into fine particles or powders [25].

Other plastic types are rarely recycled due to significant challenges [49]. These include the risk of damaging sorting equipment (like PS), as well as the low economic feasibility of their recycling processes. On the other hand, recycling of multi-layer/multi-component products is currently challenging due to the significant cross-contamination between the different materials and polymers [50]. Within this scenario, the use of various polymer combinations and the contamination by food, metal, paper, pigments, and ink complicates the recycling of polymers [51].

**Figure 6:**
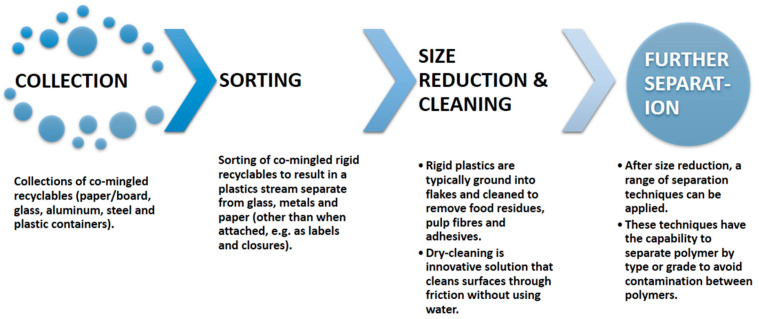
Major steps followed in plastic recycling [25].

Post-consumer recycling involves several key stages (Figure 6): collection, sorting, cleaning, size reduction, separation, and/or potentially compatibilization to minimize contamination from incompatible polymers [25].

Recycling plastic waste is becoming increasingly both economically and environmentally advantageous. Recent trends show a substantial rise in plastic waste recovery and recycling rates. Despite these encouraging trends, challenges persist. These include overcoming economic and social barriers to effective waste collection, recycling, and the use of recycled materials.

## 6. Plastic Waste Valorization/Recycling Methods

Plastic waste presents both environmental and economic opportunities through various recycling methods. These methods are primary mechanical, chemical, and energy recovery [16].

Mechanical recycling of plastics involves a multi-stage process that includes collection, sorting, cleaning, shredding, and either compatibilization or separation [51]. This process spans both primary and secondary recycling categories. The aim is to convert plastic waste into secondary materials or products without significantly altering the material’s chemical structure, ensuring that the polymer chains remain intact and are not chemically disrupted during recycling.

Chemical recycling of plastic waste involves breaking down polymer chains into their original components [52]. It commonly employs processes like chemolysis or pyrolysis; these processes involve chemically altering the polymer structure through reactions, leading to the conversion of polymers into monomers or oligomers [16]. These monomers or oligomers can then be utilized as a substitute for virgin raw materials in the production of new plastics [52].

**Figure 7:**
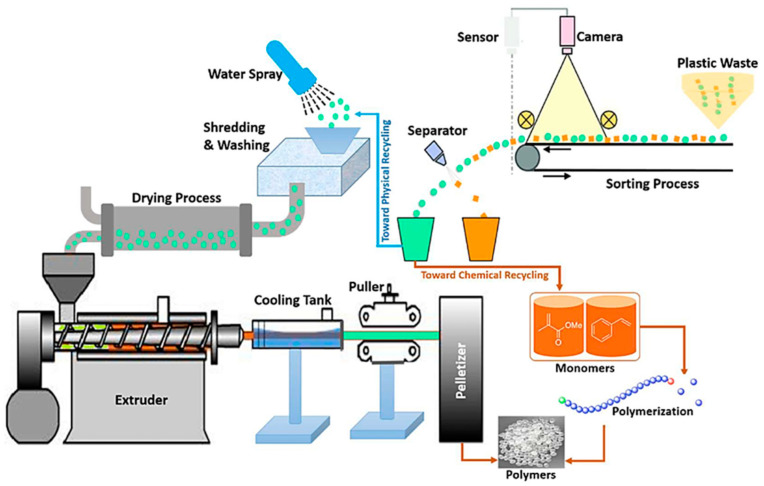
Schematic representation of plastic-waste-recycling methods: mechanical and chemical [52].

Continuous mechanical recycling can result in the production of low-quality, substandard products, a phenomenon known as downcycling [16]. Additionally, the high-chemical-input requirements and the limited applicability of chemical recycling to all plastic types make these processes potentially both uneconomical and environmentally harmful. As a result, a significant portion of these plastics ultimately end up in landfills [16]. An alternative method for managing these types of plastics is through their utilization for energy generation by incineration. Combustion typically releases harmful gases, which can be mitigated through various methods such as the addition of activated carbon, acid neutralization, and ammonia injection into the combustion chamber [48]. Figure 7 demonstrates the potential pathways for recycling plastics, encompassing both mechanical and chemical processing methods.

**Figure 8:**
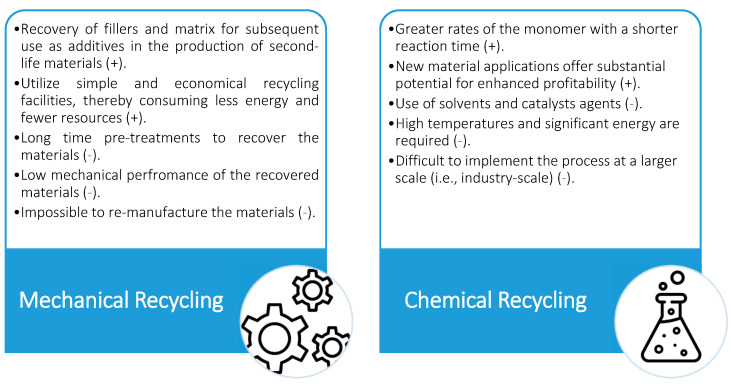
Main advantages and limitations of different recycling methods [22,52].

The advantages and limitations of different plastic-waste-recycling methods are illustrated in Figure 8.

## 7. Potential Applications of Plastic Waste

The substantial accumulation of polymeric waste from industrial and household sources has inspired researchers to explore its utilization as a solution for various challenges across diverse sectors. These waste materials are commonly employed to improve the performance of composites and to introduce novel properties to traditional materials [16].

### 7.1. Application as Soil Stabilizer

“Soil stabilization” refers to the process of systematically enhancing the engineering properties of soil to improve its stability. On the other hand, “soil amendment” encompasses a variety of techniques aimed at modifying the natural properties of soil, including physical, chemical, mechanical, biological, or integrated methods [53,54]. Recent research has explored the application of waste materials, particularly plastics, to improve soil stability. This innovative approach offers the dual benefit of significantly reducing the costs associated with soil stabilization while simultaneously addressing the environmental challenges posed by plastic waste [55].

Polyethylene terephthalate (PET) bottles are commonly used plastic containers. When shredded or cut into small pieces, PET can be integrated into expansive clay soil to enhance its structure and stability. This process improves the soil’s load-bearing capacity, making it particularly valuable in the construction industry, which has significant material consumption needs [56].

### 7.2. Application in Wastewater Treatment

Urbanization and industrialization face critical challenges in plastic waste management and wastewater treatment, driven by resource scarcity, growing energy needs, and environmental degradation. Converting plastic waste into valuable carbon nanomaterials presents a sustainable solution to address both issues [57].

The use of polymeric waste in wastewater treatment has been effectively highlighted in numerous scientific studies. A pilot study by Dorji et al. [58] found that shredded waste plastic, used as anerobic biofilter media in a wastewater treatment system, could serve as an effective alternative to traditional septic tanks, which often struggle with greywater overload and fail to meet regulatory effluent standards. Additionally, repurposing PET plastic waste into electrospun nanofiber filtration membranes have been successfully employed in microfiltration and nanofiltration, achieving a 99% removal rate of 500-nanometer beads. Functionalized mats further enhanced performance by significantly reducing biofouling and inhibiting both Gram-negative and Gram-positive bacteria [59]. Fortunately, the majority of polymeric waste, consisting of synthetic polymers such as polystyrene (PS), polyamides (PA), poly(lactic acid) (PLA), and acrylonitrile butadiene styrene (ABS), can be readily transformed into micro/nanofibers using the electrospinning technique [60,61].

The conversion of waste plastics into high-value-added carbon nanomaterials is a promising way to utilize waste plastics. Porous carbon materials derived from plastic waste can, indeed, adsorb heavy metals, dyes, and pollutants from wastewater. In this context, Gong et al. [57] addressed the limitations of single-component waste plastic research by pioneering a method to convert mixed plastic waste and clay minerals into porous carbon nanosheets (PCNSs). These PCNSs demonstrated remarkable adsorption capabilities, effectively removing methylene blue—a toxic dye—from wastewater while maintaining 91% efficiency over 10 reuse cycles, highlighting their potential for sustainable wastewater treatment. Another study explored the chemical conversion of discarded polystyrene into a polymeric flocculant, which, after sulfonation, was successfully used to filter a kaolin-containing supernatant [62]. The results were comparable to those obtained with a commercial polyacrylamide-based polyelectrolyte. Finally, Wankasi et al. [63] demonstrated the rapid adsorption of lead ions from wastewater using polyvinyl chloride (PVC) waste, achieving effective removal (99%) within 5 min at 30 °C.

### 7.3. Application in the Textile Industry

The textile industry has witnessed a significant shift from natural to synthetic fibers, primarily due to their cost-effectiveness. The growing demand for synthetic textile fibers can be attributed to several interrelated factors, including global population growth and the rising affluence of emerging economies, which drive higher consumption of textiles. Additionally, there is a significant shift from natural to synthetic materials driven by their cost-effectiveness, versatility, and durability. Increased awareness of hygiene, particularly in the context of disposable products like towels and wipes, also contributes to the demand. Moreover, the expansion of technical textiles, used in sectors ranging from healthcare to automotive and construction, further accelerates the need for these fibers [64].

Recycling waste plastics into new products for the textile industry has emerged as a critical topic in the context of environmental conservation and global sustainability. A considerable number of textile enterprises are now attempting to employ recycled plastics as primary resources for the production of synthetic fibers [65,66].

Extensive efforts have been dedicated to upcycling PET, either in its polymerized or depolymerized state. In the work of Lee et al. [64], filament fibers produced from a blend of recycled PET (sourced from used water bottles) and virgin PET demonstrated mechanical properties equivalent to those of fibers made entirely from virgin polyester.

The depolymerization of PET waste to produce new molecules for further use in the textile industry has been discussed in various scientific works. The glycolysis approach was employed to break down PET, yielding a high concentration of the pure monomer bis(2-hydroxyethyl) terephthalate (BHET), which was not only used to obtain new PET but also converted into fatty amide derivatives suitable as softeners for textile finishing [67]. In a different study, BHET produced by the glycolysis of waste PET fiber was utilized to synthesize a new cationic dyeable polyester [68]. Furthermore, a novel approach synthesized bis(6-aminohexyl) terephthalamide through the aminolysis of recycled PET (rPET) waste. Another example involved effectively producing five different types of PA66 copolyamides using the melt polycondensation method [69].

### 7.4. Other Potential Applications

Plastic recycling is primarily driven by economic factors, making the regeneration of monomers and the synthesis of high-value-added molecules particularly attractive. Vinyl polymers do contribute significantly to plastic waste, and recycling these materials is of growing importance. However, PVC recycling can be more challenging compared to other polymers, due to the potential release of harmful substances during recycling. Despite this, there is ongoing research to improve the recycling processes for vinyl polymers and to find ways to regenerate valuable monomers or high-performance products from them.

For polypropylene, the most efficient method of converting it back to propylene involves non-pyrolytic approaches, particularly ionization. Inductively coupled plasma has demonstrated the ability to transform polypropylene into a gas mixture containing 94% propylene [70].

In contrast, the chemical inertness of polyethylenes poses significant challenges for their degradation through low-energy processes. A novel tandem catalytic system has been developed for polyethylene depolymerization, yielding various n-alkanes and polyethylene waxes [71].

Polystyrene, another challenging vinyl polymer, has motivated efforts to identify alternatives to conventional recycling methods. For instance, waste polystyrene can undergo sulfonation to produce polyanions, which function as effective flocculating agents [72]. Additionally, leveraging the solubility of polystyrene enables the conversion of waste polystyrene into pure microcellular foams with controlled pore sizes [73].

The potential uses of plastic waste for nanomaterials production have been the subject of investigation in numerous research endeavors. Discarded polymers were employing for the production of metal-based electrocatalyst nanoparticles from polyvinyl chloride, photoluminescent carbon nanoparticles from polypropylene and polylactide, and superabsorbent nanocomposite from polystyrene [74,75,76,77,78]. The successful development of a cost-effective technology has been achieved by researchers, enabling the conversion of waste PP into superhydrophobic nanostructured spheres by carrying out a chemical treatment procedure. The water contact angle measurement of these spheres was determined to be 164°, which is similar to the one observed on the surface of lotus leaves. Superhydrophobic materials enable numerous applications, including, but not limited to, coating, self-cleaning, and water–oil separation processes [79].

The conversion of waste plastics into carbon nanostructures of high value has also emerged as a promising avenue. The manufacture of carbon nanotubes, a process that frequently requires energy- and resource-intensive production processes, was conducted, utilizing waste plastics derived from either homopolymers or blends, such as polystyrene, polypropylene, and polyethylene [80,81,82]. Furthermore, combinations of various plastic materials, namely vinyl polymers (PS, PP, PE, PVC) and polyesters, were effectively used in the fabrication process of porous carbon nanosheets [57,83].

The utilization of polymer waste as a resource for manufacturing high-energy storage supercapacitors has seen significant growth in recent years. This strategy involves controlled combustion, which produces carbon compounds with exceptional conductivity, and depolymerization, which breaks down polymer chains to yield novel compounds with reduced molecular weight. [84]. Extensive research is currently being conducted on several components of energy storage systems, namely cathode, anode, binder, and electrolyte. Numerous organic materials are being explored as potential candidates for fulfilling these specific tasks [85,86,87,88,89].

## 8. Application of Recycled Plastic Waste in Construction

**Figure 9:**
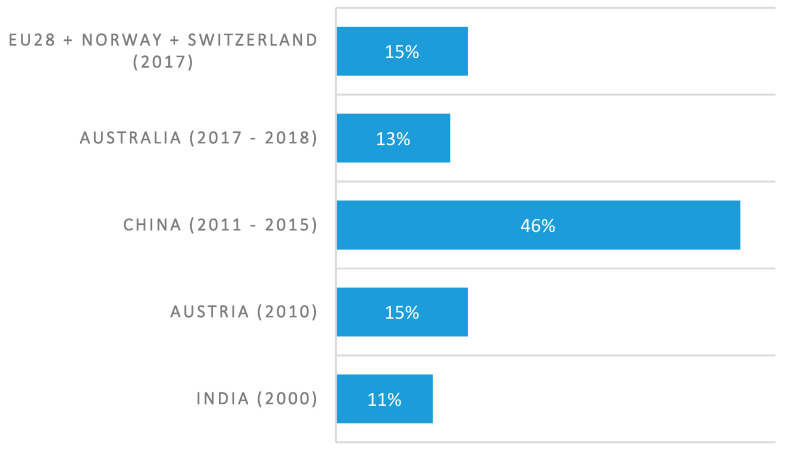
Recycled plastic waste in construction sector [93].

In recent years, there has been a growing interest within the building and construction sectors to explore the utilization of recycled plastic waste as a feasible material (Figure 9). Based on existing experimental studies, it has been demonstrated that plastics can be effectively employed either independently or in combination with other materials, at different percentage compositions, to create a wide range of building and construction materials [90]. These materials encompass bricks, blocks, tiles, asphalt, bitumen, geo-synthetics, door panels, insulation materials, and cementitious composites [90].

This trend helps address environmental concerns by reducing plastic waste, minimizing the use of natural aggregates, and lowering carbon emissions from conventional material production and transportation [91]. The construction industry, indeed, ranks among the largest energy and non-renewable resource consumers globally. This sector accounts for approximately one-third of the world’s final energy consumption and nearly a quarter of direct CO_2_ emissions [92].

In the construction industry, the demand for cement and non-renewable raw materials, such as natural sand, has risen due to global urbanization. As cement consumption increases, the associated CO_2_ emissions from its production are becoming a growing concern [94]. According to current estimates, global carbon dioxide emissions from cement and fossil fuels climbed by 1.1% in 2023 relative to 2022, setting a new high of 36.8 billion tons of CO_2_ (GtCO_2_) [95]. In addition, significant quantities of non-biodegradable plastic waste are produced and end up in the environment. Waste dumped in landfills consumes already limited land resources and discharges dangerous toxins into the groundwater, air, and soil [96]. One potential solution to mitigate these issues is recycling of plastic waste in the construction sector. Recycled plastic waste is, indeed, widely recognized in numerous research studies for its utilization in construction to decrease the consumption of non-renewable raw materials [91]. This win–win approach not only helps reduce the volume of plastic waste in the environment but also offers eco-friendly alternative materials.

As previously discussed, plastic waste is classified as thermoplastic and thermoset, each exhibiting distinct properties. Given their known distinct properties, the recycling of thermoplastic and thermoset waste plastics necessitates different application approaches. Thermoplastics find utility in applications demanding flexibility, toughness, and impact resistance, while thermosetting is favored in applications demanding high strength, durability, and heat resistance. Moreover, they are ideal where high dimensional stability is required. Considering their potential attributes, the incorporation of thermoplastic and thermoset plastic waste in construction applications offers significant advantages.

**Figure 10:**
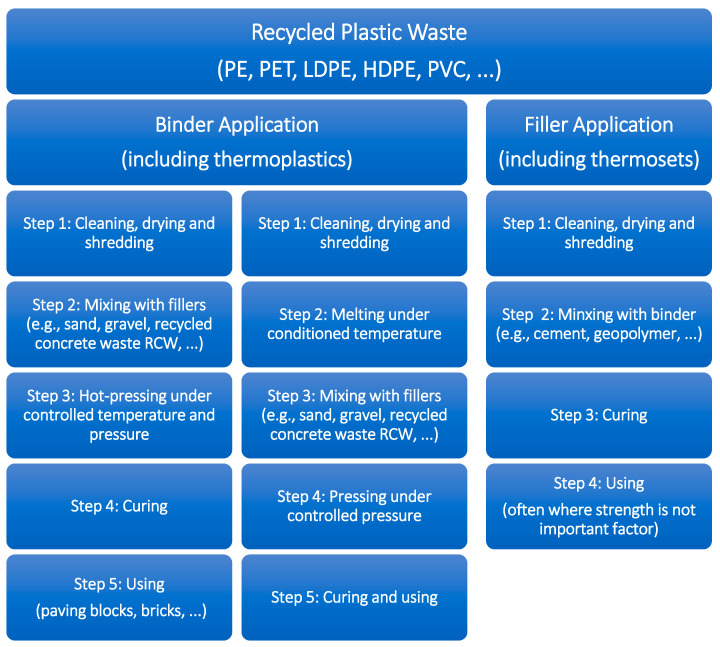
Diagram showing the common steps for recycling plastic waste in construction applications.

The different types of waste plastics and their applications in construction materials were recently revised by Hamada et al. [97]. Figure 10 shows the common steps followed in the literature during the process of recycling plastic waste for application in construction. Additional details were mentioned by Mailto et al. [91].

Employing recycled thermoplastic waste as a binder offers several advantages. Thermoplastics exhibit desirable properties such as softness and flexibility when heated, facilitating easy molding and shaping. Additionally, they are lightweight and can be recycled many times. However, their primary drawbacks include limited resistance to heat and chemicals. In contrast, recycled thermosetting plastic wastes are well suited for use as filler due to their inherent rigidity and hardness even when heated, making them difficult to mold and shape. However, they exhibit superior resistance to heat and chemicals, along with exceptional strength and durability. These characteristics provide a roadmap for the applications of thermoplastic and thermoset plastic waste in the construction sector. Figure 10 provides a brief overlook of the possible alternatives for plastic recycling in concrete, highlighting the pros/cons of the three analyzed alternatives.

### 8.1. Recycled Plastic Waste as Filler

**Figure 11:**
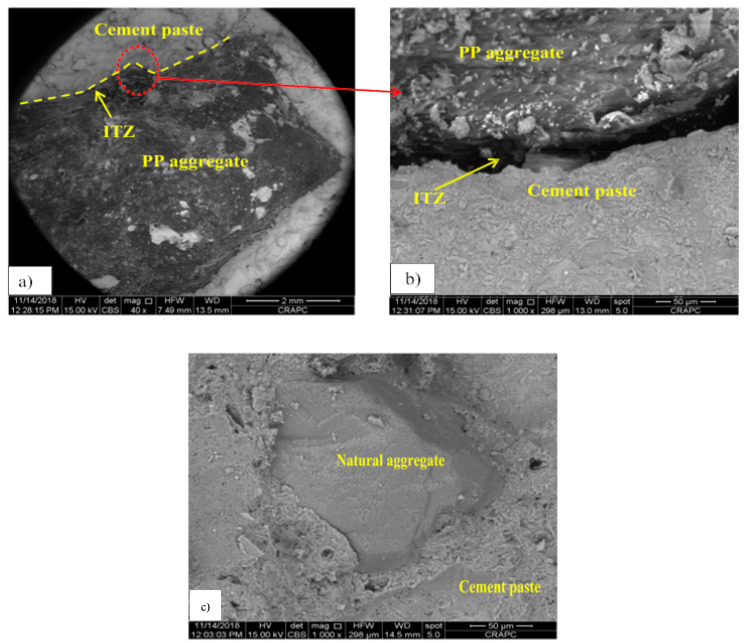
SEM images of plastic aggregates and cement paste: (**a**) interfacial transition zone (ITZ), (**b**) magnified area, (**c**) reference concrete. Image reproduced with permission from [100]. Copyright Elsevier 2020.

Besides the plastic waste pollution, another environmental challenge arises from the unsustainable widespread use of sand [98]. According to UNEP (United Nations Environment Program) [99], after water, sand is the second most utilized resource on Earth. It is typically obtained by dredging rivers, excavating coastal areas, and mining. The estimated 50 billion tons of sand that is extracted annually for construction purposes alone would be sufficient to construct a nine-storey wall around the entire planet [99]. Many methods have been suggested to reduce the consumption of new sand and promote the circular economy: recycling construction materials from demolition sites and utilizing ore-sand, a by-product of mineral processing designed for construction and industrial applications. Ore-sand can potentially provide an alternative source of sand while reducing the production of mine tailings. Numerous researchers have explored the impacts of incorporating plastic as a partial replacement for sand in cement concrete (Figure 11). In general terms, it may be stated that concrete with low plastic stiffness will exhibit a lower elastic modulus, and similarly, low-strength plastic will have a greater impact on the final strength of the concrete. This review examines the key factors influencing how recycled plastic, when used as a sand substitute, affects concrete properties.

Due to the inadequate bond between plastic particles and the surrounding matrix, which weakens the interfacial transition zone, adding plastic to a concrete mix generally leads to a reduction in compressive and tensile strengths. SEM analysis of cement-based concrete in the study of Belmokaddem et al. [100] demonstrates a wider transition interfacial zone and reduced adhesion between polypropylene (PP) aggregate and cement paste (large air bubbles were observed), in contrast to natural aggregate (Figure 11). To enhance mechanical performance, numerous studies recommend chemical or physical surface treatments of plastic fillers before their use as a partial or full replacement for natural aggregates in concrete.

Table 2 shows physical properties of natural sand compared to some plastic sand.

Almeshal et al. [101] investigated whether plastic PET could replace a portion of the aggregates in concrete. They made six different mixtures with various amounts of PET grinded bottles (0–50%) and tested their physical and mechanical properties. The performances of both fresh and hardened concrete were analyzed in terms of properties such as workability, unit weight, compressive strength, flexural strength, tensile strength, ultrasonic pulse velocity, and fire resistance. According to the experimental findings, high PET content lowers mechanical properties and fire resistance while decreasing the unit weight of the concrete. Nevertheless, the authors indicated that plastic waste can be disposed of in certain ratios and utilized efficiently in industrial applications that do not involve high compressive strength, such as in pavements and non-structural elements.

**Figure 12:**
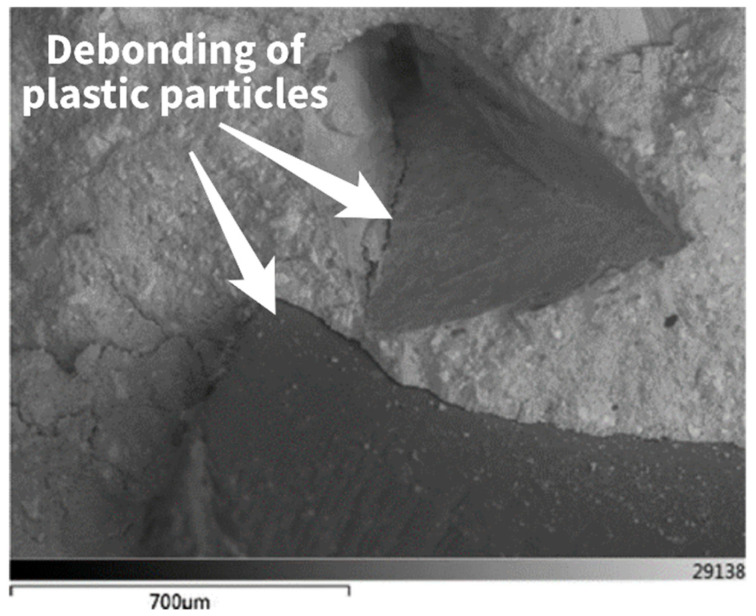
SEM image showing debonding of plastic particles from the surrounding matrix at failure. Image reproduced with permission form ref. [102] Copyright Elsevier 2018.

The study by Thorneycroft et al. [102] demonstrated that recycled plastic waste (PET) could be used in structural concrete mixtures, potentially reducing the usage of 820 million tons of sand per year at a 10% replacement ratio by volume. Reducing sand demand from the construction sector would help mitigate the negative impacts of sand dredging in countries where large quantities of sand are extracted annually. The study found that the most effective plastic aggregates for application in concrete should have a rough surface, an irregular shape, be small enough to avoid significant failure surfaces, and possess a grading similar to the sand they replace. Strength reduction, primarily caused by poor bonding (Figure 12), can be minimized to acceptable levels with an optimized mix design. However, before plastic can be widely adopted in moderate- to high-strength structural concrete, further research is needed to enhance the bond between the matrix and the plastic, particularly through chemical treatments.

The study of Akinwumi et al. [103] explored the use of recycled plastic waste (PET) in cement-stabilized soil as a road pavement layer material. The pozzolanic reaction, involving the interaction of amorphous SiO_2_ and Al_2_O_3_ with Ca(OH)_2_ from cement hydration, likely contributed to strengthening the soil by enhancing the bonding between soil particles, through cementitious compounds. However, the addition of plastic waste to the cement-stabilized mixture reduced the strength characteristics of the mixture. However, the soil with 10% cement and 2% plastic waste performed better than the other mixtures, making it suitable for use as a layer material in road pavement construction [100].

One effective method for assessing the uniformity, consistency, and overall quality of concrete is an ultrasonic pulse velocity (UPV) test. This test not only helps identify imperfections, such as cracks or voids, but is also useful for evaluating the level of compactness. If the UPV value of a concrete specimen falls within the range of 3660–4575 m/s [104] or, according to other references, 4100–4500 m/s [105], it is considered to be of high quality. Numerous studies [102,105,106] have noted that increasing the plastic ratio leads to a decrease in UPV, being primarily influenced by the volumetric composition of the concrete and the lower elastic properties of plastic compared to natural aggregates. As a result, substituting sand with plastic inherently lowers the UPV [101]. According to Albano et al. [106], the morphology of the plastic aggregates within the matrix significantly influences the properties of cement-based composites, directly impacting the porosity of concrete. The presence of plastic particles creates air voids, which reduce the transmission of ultrasonic waves. As ultrasonic waves traverse various materials—such as concrete, plastic aggregates, and air voids—they experience partial reflection and decreased transmission, resulting in reduced velocity [100,106]. Although UPV primarily measures material compactness and density, lower UPV values may indirectly suggest changes in acoustic insulation properties due to increased porosity [107]. For example, a 50% substitution of sand with PET was associated with a 57% reduction in sound velocity, suggesting an impact on acoustic insulation properties [101].

In addition to potential acoustic insulation, the thermal insulation properties of the composites also improve with the use of plastic waste as partial or full replacement of natural aggregates [108,109,110]. This enhancement is attributed to the low thermal conductivity of plastics, such as LDPE (0.35 W/(m·K)), PP (0.23 W/(m·K)), and HDPE (0.43 W/(m·K)) at 25 °C [111]. The study of Aciu et al. [112] demonstrated that incorporating PVC waste into mortar compositions significantly reduces thermal conductivity. Specifically, replacing 25% of the sand with PVC waste decreased the thermal conductivity coefficient by 65% compared to standard mortar, while doubling the replacement amount led to a 73% reduction.

Moreover, the use of plastic aggregates may be particularly suitable for concrete pavement due to the low heat capacity of plastic waste. This characteristic allows the pavement to heat up during the day but cool down rapidly at night, helping to mitigate the urban heat island effect during nighttime [100]. On the other hand, incorporating recycled plastic waste into the construction sector may offer a valuable solution to energy balance challenges and significantly enhance the thermal efficiency of buildings [100]. Mendes et al. [113] found that thermal conductivity can be estimated based on UPV, noting a stronger correlation between UPV and thermal conductivity than between thermal conductivity and specific gravity. As previously mentioned, indeed, the speed of ultrasonic waves is affected by both the elastic characteristics of the medium and the composition of the concrete.

Concrete has historically exhibited superior fire resistance compared to steel, primarily due to its non-combustible properties [101]. Aciu et al. [112] demonstrated that standard mortar falls into fire reaction class A1 (non-combustible) since it contains no organic materials. This characteristic distinguishes concrete as a material capable of forming effective thermal barriers that resist high temperatures and slow the spread of fire. As a result, fire resistance testing is often not required for concretes and mortars, particularly when using conventional aggregates. However, it is crucial to investigate the behavior of plastics, especially thermoplastics, when incorporated into concrete, as they may respond differently under fire conditions. The fire resistance of recycled plastic waste composites can be assessed through experimental tests involving exposure to direct flames and thermal loads. In a study conducted by Bodnarova et al. [114], several steps were identified to determine the fire resistance level of materials: (i) before flame exposure, the physicomechanical and other properties of all specimens were tested, including density, compressive strength, flexural strength, moisture content, and surface appearance; (ii) during exposure, specimens were visually observed, and any changes were recorded. The exposed surface was photographically documented before thermal load and every 10 min intervals, while a thermocamera monitored the temperature development. After cooling, the specimens were again visually inspected for cracks, and the maximum depth of the spalled area was measured.

**Figure 13:**
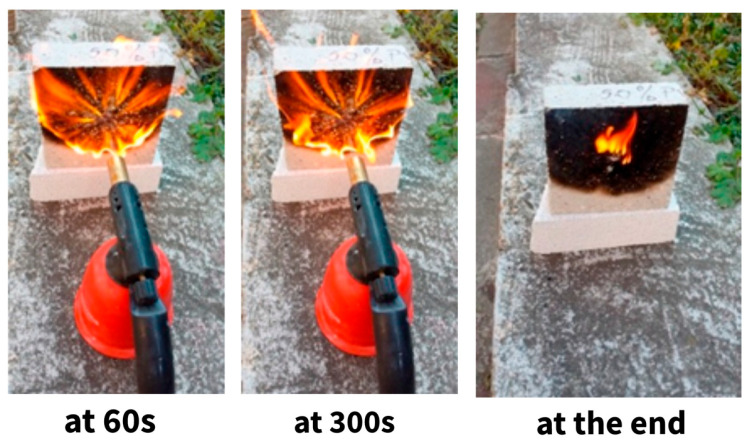
Fire behavior of mortar with 50% PVC plastic after 60 s, 300 s, and after the removal of the flame. Image reproduced with permission from [112]. Copyright Elsevier 2018.

Research has shown that the fire resistance of plastic–concrete composites can be estimated using direct flame tests generated by a gas lamp [112,114]. Some plastic-based formulations may ignite quickly and release toxic black smoke. However, the flames typically affect only a localized area around the ignition point, limiting their spread and preventing the material from igniting beyond that zone. Once the applied flame is removed, indeed, the ignited particles continue to burn until all combustible material is consumed, at which point the fire extinguishes (Figure 13).

Lastly, Steyn et al. [115] investigated the effect of replacing sand with up to 30% recycled LDPE particles, finding that when low substitution ratios are adopted, good concrete workability and mechanical performance can be achieved, allowing concrete to be used for various applications, including structural ones. The only limitations they identified were excess water and poor bonding at the interfacial transition zone (ITZ) between the plastic particles and the cement matrix, which negatively affect concrete durability, among other factors. Specifically, two key transport properties—water sorptivity and the oxygen permeability index—showed reduced durability when high plastic content was used, consistent with the observed increase in concrete porosity. However, Saikia and de Brito [116] highlighted the opposite behavior, reviewing many works where the authors found that different types of plastic waste as aggregates can improve the permeability of concrete, leading to enhanced durability against aggressive attacks and frost resistance.

### 8.2. Recycled Plastic Waste as Binder

One approach to incorporating recycled plastic waste into construction is the melting process. This method involves collecting and thoroughly cleaning plastic waste, such as bottles, bags, and sheets. The cleaned plastic is then heated to high temperatures until it melts, allowing it to be combined with materials like sand, sawdust, or other construction components. The resulting composite is molded into products like bricks, tiles, or asphalt elements, which are then cooled and solidified to form durable building materials. These materials can be used for a variety of purposes, including road construction and roofing applications. Additionally, their thermal insulation properties enhance energy efficiency and contribute to the sustainability of construction projects

This method is gaining attention primarily because thermoplastics represent the most prevalent category of plastic waste. A significant portion of plastic waste, indeed, comprises various types of thermoplastics, including polypropylene (PP) at 21%, low-density polyethylene (LDPE) and linear low-density polyethylene (LLDPE) at 18%, polyvinyl chloride (PVC) at 17%, and high-density polyethylene (HDPE) at 15%. Additionally, other plastic types include polystyrene (PS) and expanded polystyrene (EPS) at 8%, polyethylene terephthalate (PET) at 7% (excluding PET fibers), as well as thermosetting plastic polyurethane [33].

The study by Thiam et al. [117] investigated the properties of mortars using melted plastic waste binders, specifically high-density polyethylene (HDPE) and low-density polyethylene (LDPE), for construction applications. Initial tests revealed that when the plastic content is below 40%, the mortar exhibits uneven distribution, resulting in a heterogeneous mixture where some sand particles remain uncovered by the plastic binder. On the other hand, when the plastic content exceeds 70%, the mortar becomes excessively fluid, leading to rapid settling of the sand grains and causing segregation and inconsistency during the casting process. Therefore, the optimal plastic content in the dry sand is re-commended to range from 45% to 65% of the total mass

The results revealed that the new material maintained a compressive strength of 5–18 MPa and a splitting tensile strength of 1–5 MPa across different plastic contents and testing periods. Additionally, the incorporation of melted plastic led to lower water absorption through both immersion and capillarity. Furthermore, the study found that the melting process and the interaction of plastic with inorganic materials (such as sand and gravel) enhanced the thermal stability of the plastic mix compared to pure plastic waste.

**Figure 14:**
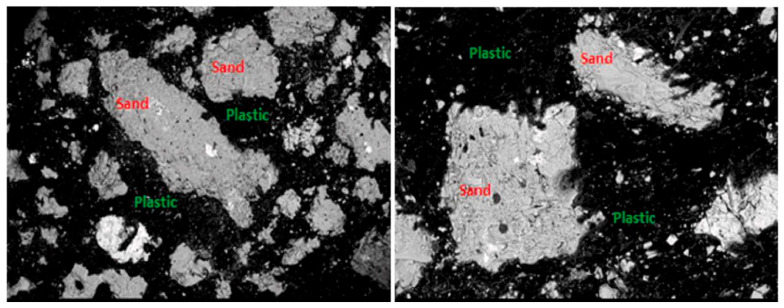
SEM images: sample with 60% plastic (magnified 30× (**left image**) and 100× (**right image**)). Image adapted from [117]. Copyright Elsevier 2021.

Thiam et al. [118] conducted a further, separate study, where they used SEM analysis to examine the binding of granular mineral materials (such as sand and gravel) with melted plastic (HDPE and LDPE). The results indicated an evolution in void formation: during the initial stage of curing, the presence of pores was higher, while during the polymers solidification they are filled thanks to the change in volume of the matrix, leading to a denser structure. The ITZ morphology differs significantly from that of a virgin concrete with cement binder. Additionally, an increase in the proportion of melted plastic in the concrete improved its mechanical strength thanks to a reduction in the porosity. SEM images in Figure 14 show the composition of the mortar sample with 60% plastic and HDPE/LDPE ratio of 40/60.

In another study, Thiam et al. [119] conducted an experiment to investigate the effects of curing conditions and granular material fineness on the mechanical properties of the mortar with a melted plastic waste binder (HDPE and LDPE). The results indicated that curing methods significantly influenced compressive and splitting tensile strengths. Samples cured in air at ambient temperature exhibited higher strength than those cured in water, where strength decreased due to increased porosity and a coarser pore structure caused by the faster cooling rate during water curing. SEM analysis revealed that lower-strength samples had higher absorption rates and coarser pore structures. Additionally, the mechanical performance was affected by the grain size distribution of mineral aggregates. Introducing 30% granular material (sand and gravel) within the 2–4.75 mm range to a sand matrix with grain sizes below 2 mm significantly enhanced compressive and splitting tensile strengths. This improvement was attributed to increased packing density of mineral aggregates, which led to higher hardened density in the samples. In essence, the added granular material fills the gaps within the material, resulting in greater density and improved mechanical performance upon hardening.

Sakai et al. [120] conducted a pioneering study that introduced an innovative recycling approach, emphasizing the efficient production of concrete while preventing the segregation of gravel, sand, or other components. Their method involves the recycling of concrete waste through compaction, eliminating the need for additional raw materials. The researchers crushed the demolished concrete, and then milled it into a powder form. Subsequently, the powdered concrete was compacted under high pressure to create new concrete samples. This technique offers two significant benefits: a rapid production speed and a closed-loop system for the utilization of concrete materials.

**Figure 15:**
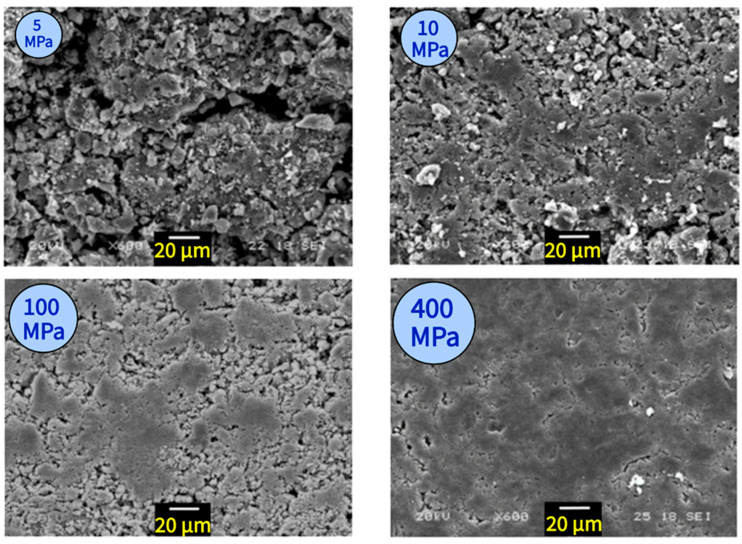
SEM observations of hardened cement paste under various compaction pressures. Image reproduced with permission from [120]. Copyright J-Stage 2016.

The process of concrete consolidation or concrete compaction involves removing trapped air (voids and air pockets) from the plastic matrix of the concrete [121]. This leads to a more compact and stronger concrete mixture with lower permeability, resulting in increased durability [121]. Figure 15 shows the concrete after compaction at different applied pressures. When a pressure of 5 MPa was applied, the particles underwent minimal change and just made contact with each other. However, when the pressure exceeded 10 MPa, the particles significantly deformed, filling the spaces between them. As a result, the boundaries separating the particles became indistinct.

**Figure 16:**
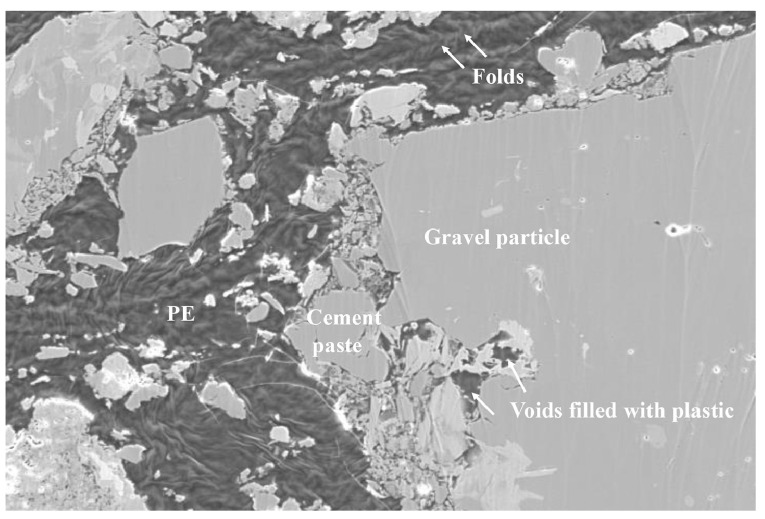
SEM image (magnification 2000×) of compacted plastic concrete (25% plastic to concrete ratio). Images reproduced with permission from [96]. Copyright Elsevier 2021.

This research revealed a significant challenge in achieving the required pressure for sufficient compact strength. This challenge is not only energy-consuming, as it can reach up to 100 MPa, but also time-consuming, with the compaction process lasting for 10 min. These factors pose practical production difficulties. Wei et al. [96] conducted a study that introduced a unique approach to address the aforementioned issues. Their method involves the inclusion of plastic in concrete recycling through high-temperature compression (Figure 16). The findings indicated that plastic concrete compacted at a maximum pressure of 50 MPa exhibited superior bending strength compared to conventional concrete. In comparison to the test conducted by Sakai et al. [120] using a pressure of 100 MPa, the inclusion of plastic waste powder significantly reduced the compaction pressure required for forming, thereby enhancing the energy efficiency of the recycling process. Moreover, the successful formation of plastic concrete was strongly influenced by an optimal molding temperature, which was set below the melting point of the plastic material. The hot-pressing process played a crucial role in facilitating the bonding between the plastic and concrete powders. However, it should be noted that excessively high temperatures could lead to thermal aging of the plastic, resulting in a decline in the performance of the compacted plastic concrete.

**Figure 17:**
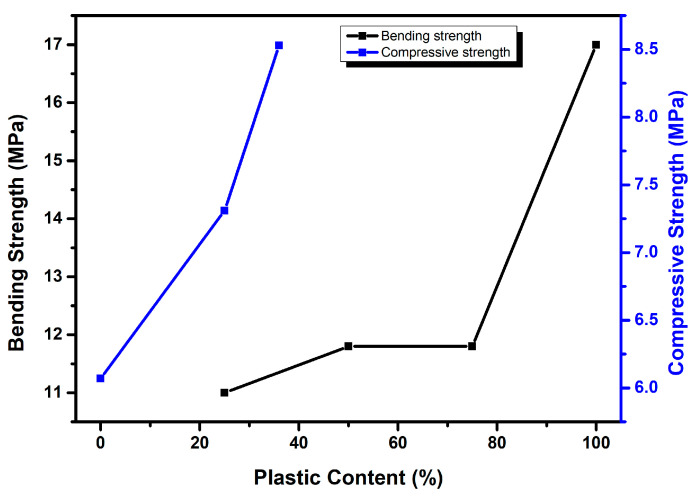
Graph showing the evolution of bending strength of compacted concrete with increasing melted plastic content (black) [96] and compressive strength of recycled plastic waste units (blue) [123].

In contrast to using non-melted plastic waste as aggregates in fresh concrete, the strength of the compacted concrete did not diminish as the plastic content increased (Figure 17). The strong adhesion between the plastic and concrete powder during the hot-pressing process may have led to this outcome [96]. Jo et al. [122] observed a similar phenomenon and discovered that the strength of polymer concrete, made with a resin derived from recycled PET and recycled aggregate, increases as the resin content increases. However, they also noted that once a certain resin content is reached, there is no significant change in strength. Figure 16 illustrates the notable absence of voids within the plastic–concrete interfacial region, with even the minor voids being filled by plastic particles. Under the conditions of high temperature and high pressure, the plastic melted and then smoothly filled the empty spaces; therefore, hot-pressing was used to eliminate the poor bonding associated with the conventional method [96].

A study was conducted by Agyeman et al. [123] to explore the potential of utilizing plastic waste (thermoplastics) as a binding material in the production of paving blocks. During the experiment, varying quantities of melted recycled plastic waste were poured into a mold in three layers, resulting in composite paving units. Subsequently, the units were compacted manually using a tamping rod. Through various laboratory tests, they found that the specimens with plastic had higher compressive strength compared to the control specimens without plastic (Figure 17). This might be attributed to the stronger adhesive bond between the plastic waste’s surface area and the nearby aggregate particles [124]. Compared to the bond formed by the cement binder, it appears that the plastic waste binder and aggregates have a stronger binding. In addition, it was shown that utilizing plastic waste resulted in lightweight materials by reducing density, increasing ductility, and improving workability [124]. Furthermore, the manufacturing process benefits from significant time savings when using molten recycled plastic waste units, as they achieve over 80% of their final strength within a single day.

An essential quality of concrete is the capacity to absorb water, which is a key transport characteristic that serves as a critical indicator for assessing and predicting its durability [125]. Given that the pore structure of concrete is known to significantly impact its durability, research often focuses on developing simple tests to characterize this structure and establish basic standards for concrete durability. In this regard, water absorption through immersion is considered a key parameter [126].

**Figure 18:**
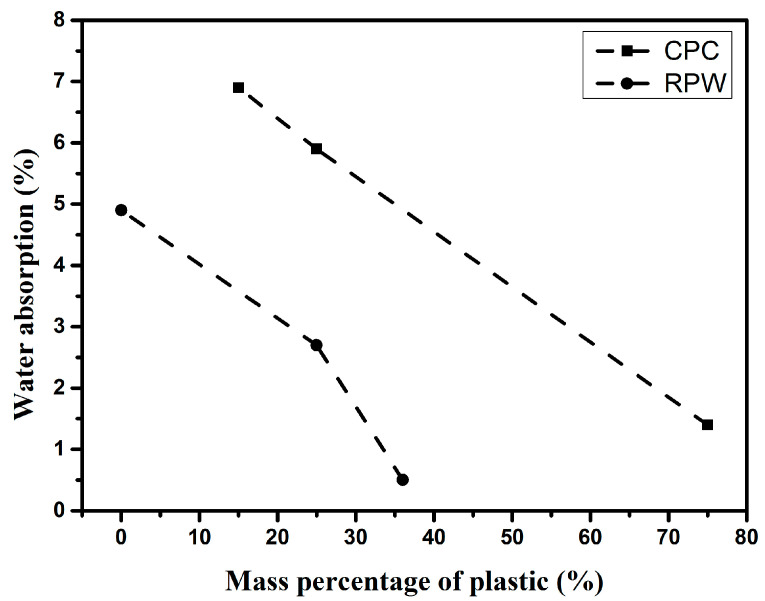
Evolution of water absorption with plastic content, case of recycled plastic waste units (RPW) [123], and compacted plastic concrete (CPC) [96].

The findings showed that the cement concrete units (with no plastic), which were determined to be hydrophilic, absorbed more water than the recycled plastic waste concrete (hydrophobic) [96,123]. This shows that the plastic content contributes to reducing water absorption of the concrete (Figure 18). Experimental study on LDPE by Youssef et al. [127] showed that the water absorption of the plastic ranged between 0% and 3% during 2 weeks of test with small swelling range between 0% and 2%. High water absorption can restrict the use of concrete in various construction applications. For concrete paving blocks, structural elements, and exterior building bricks, the water absorption should be less than 7% [128].

## 9. Pros and Cons of Using Plastic Waste in Constructions

In summary, the application of recycled plastic waste in the construction sector offers a promising strategy for reducing plastic pollution, diverting waste from landfills, and promoting natural resource conservation. Nevertheless, performance should be carefully evaluated on a case-by-case basis. Table 3 summarizes the general advantages and disadvantages of construction materials incorporating plastic waste, based on the three main methodologies described herein, and provides relevant examples for each type of composite.

Moreover, several environmental and economic issues must be considered when using plastic waste in construction:

(i) Long-term performance under different environmental conditions, including extreme weather conditions and harsh conditions (acidic or saline environment), which may accelerate the degradation over time and require frequent repairs [129].
(ii) The environmental impact due to degradation: The composites may contribute to long-term environmental pollution if not properly managed at the end of their life cycle, as they may degrade slowly in natural environments and release both additives (e.g., plasticizers, stabilizers) and microplastics. Pavements and other materials undergo mechanical abrasion, leading to the release of microplastics even before they start to degrade [130].
(iii) Health and safety concerns: The handling of certain types of plastic waste (especially when heated or melted) may release harmful fumes or chemicals, posing health risks to workers involved in the manufacturing process [131].
(iv) Variability of plastic feedstock and presence of impurities: The properties of recycled plastic waste can vary depending on the source and type of plastic. This variability can make it difficult to maintain consistent quality and performance in construction materials [132].
(v) Cost of processing and treatment: The processing of plastic waste (e.g., sorting, cleaning, melting) can be energy-intensive. Additionally, pretreatment methods, such as surface modifications, may be necessary to improve compatibilization with other common construction materials, complicating the production and further increasing the cost [133].

These considerations highlight the need for further research and innovation to overcome the challenges associated with using recycled plastic waste in construction, ensuring both environmental sustainability and material performance. To mitigate this risk, it is crucial to consider the long-term behavior of plastic-based construction materials, monitor their degradation, and explore innovative ways to enhance their stability and prevent fragmentation into microplastics.

## 10. Conclusions

Among the various solutions for plastic recycling and valorization, the use of recycled plastic waste in the construction sector offers several key benefits. Recycled plastic waste can be applied either as a filler (e.g., aggregates) or as a binder. The properties of the resulting material depend on many factors, including the formulation, the content, and type of the recycled plastic waste (thermoplastic or thermoset) and the production process.

When used as a filler, recycled plastic waste can replace non-renewable natural resources, potentially reducing the consumption of 820 million tons of sand annually at a 10% volume replacement ratio. As the recycled plastic waste content increases, the mechanical properties and fire resistance of the composites generally decrease, while their weight is reduced. The reduction in strength is primarily due to the poor bonding between the plastic aggregates and the cement matrix. Therefore, recycled plastic waste can be effectively used as a filler in specific ratios for industrial applications where strength is not a critical factor, such as in pavements and non-structural elements. Additionally, incorporating recycled plastic waste as a filler in the construction sector offers a valuable solution to energy balance challenges, enhancing both thermal and acoustic efficiency in buildings.

Thermoset plastic waste is generally preferred over thermoplastic waste as a filler for partial or full aggregate replacement. Thermosetting plastics are rigid, heat-resistant, chemically durable, and strong, making them ideal for such applications. However, many studies suggest that chemical or physical surface treatments may be needed to improve the mechanical performance of plastic fillers. It is also crucial to assess the behavior of plastics, particularly thermoplastics, which are more prone to combustion compared to traditional construction materials. In this context, embedding the plastic filler into the composite material may offer a suitable strategy to prevent environmental exposure, reduce the risk of faster degradation, and limit the release of harmful substances.

Recycled plastic waste is often melted and combined with materials like sand to serve as a binder for creating innovative composites. This method is gaining attention, primarily because thermoplastics make up the largest category of plastic waste. The optimal plastic content in these composites is generally recommended to range from 45% to 65% of the total mass. Incorporating melted recycled plastic waste enhances the ductility of the composites, reduces water absorption, and improves durability. In addition, concrete with melted plastic exhibits superior mechanical performance relative to mixtures containing unmelted plastic filler. Furthermore, to efficiently produce concrete without segregation of components and with fewer pores, hot compaction is employed. This process results in a denser and stronger mixture with lower permeability. Hot pressing is particularly important for improving the bonding between plastic and other materials. Under high temperature and pressure, the melted recycled plastic fills the voids more effectively, and hot pressing is especially effective in overcoming poor bonding issues commonly encountered with traditional methods.

Overall, the use of recycled plastic waste in construction materials offers significant benefits as a sustainable solution to both plastic waste management and resource conservation. However, further research, including Life Cycle Assessment (LCA), is essential to address challenges related to mechanical performance, fire resistance, long-term durability, and the environmental impact throughout the material’s lifecycle. Additionally, it is still crucial to implement strategies to avoid the potential release of plastic fragments and pollutants, ensuring that these environmental concerns are minimized.

## Figures and Tables

**Table 1:** Polymers of large usage [16,25].

Polymer Symbol	Polymer Chemical Structure and Applications	Recycling Code	Production by Polymer Type (EU-2022) [26]
Polyethylene Terephthalate (PET)	Fibers, 3D printing filaments, bottles for beverage, electrical appliance components, etc. 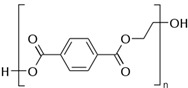	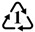 Easy production process; recyclable and reusable; high calorific value; inexpensive	5.0%
High-Density Polyethylene (HDPE)	Flexible and rigid pipes, fibers, milk jug, ballistic plates, etc. -[CH_2_-CH_2_-]_n_-	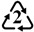 Durable; withstands most solvents; can be recycled multiple times	8.7%
Polyvinyl Chloride (PVC)	Electric cables, pipes, weather-resistant coats, sheets, etc. -[CH_2_-CHCl-]n-	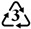 Durable and resistant to harsh conditions; leaches chemicals over time; recycled PVC has lower market value; challenges in waste collection	9.1%
Low-Density Polyethylene (LDPE)	Bags, houseware, trays, containers, etc. -[CH_2_-CH_2_-]_n_-	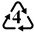 Soft, lightweight, and less toxic; difficult to fully recycle; challenges in waste collection	13.4%
Polypropylene (PP)	Containers, electrical cables, medicals devices, nonwoven fabrics 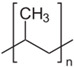	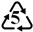 Tough and lightweight; heat-resistant; durable; easily recycled in semi-rigid form but more difficult to recycle in film form.	15.4%
Polystyrene (PS)	Packaging, containers, rigid panels, etc. 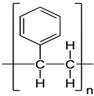	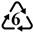 Hard to recycle; leaches chemicals when heated; requires separation from other waste streams; inexpensive	5.4%
Polyamide (PA) Polycarbonate (PC) Polyacrylonitrile (PAN) Polyurethane (PU)	Compact disks, medical devices, baby bottles, foams, etc.	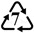 Almost never recycled; require separation from other waste streams	Other fossil-based plastics 23.3%

**Table 2:** Physical properties of some types of sand [100].

Properties	Natural Sand	PVC Sand	PP Sand	HDPE Sand
Density (g/cm^3^)	2.68	1.40	0.88	0.96
Grain size (mm)	0–3	0–3	0–3	0–3
Shape	Angular	Angular	Angular	Angular

**Table 3:** Advantages and disadvantages of conventional construction materials versus materials with plastic waste incorporated.

Composite Material	Main Features	Advantages	Disadvantages
Concrete with plastic particles as filler—sand substitution	- Optimal range: up to 10% with few impacts on strength and durability - PET, LDPE, HDPE, PVC investigated	- Reduction in sand consumption - Reduction in unit weight - Reduction in thermal conductivity - Reduction in costs and impacts.	- Reduction in workability (if flaky particles are used) - Reduction in mechanical strength (irrespective of the type and amount of plastic) - Increased porosity - Reduction in ITZ strength - Reduction in durability-related performances
*Example* Lightweight recycled plastic aggregate concrete [108].	- Mostly mixed PE. - Recycled plastic waste produced in the shape of granules, fibers, and flakes - Small size of plastics (5–10 mm). - Fiber plastic about 0.5 mm and the length in the range of 3–10 mm.	- Lightweight: decrease in unit weight with plastic content. - Thermal insulation: decrease in thermal conductivity by 35–65% compared to the control concrete. - Results: using 100% recycled plastics led to unit weight of 1500 kg/m^3^ and a compressive strength of 17 MPa. - Results: using 25% recycled plastics led to unit weight of 2000 kg/m^3^, and a compressive strength of 35 MPa. - Thermal conductivity of the control concrete is 1.7 W/mK while it is around 1.1–0.5 W/mK for the plastic–concrete.	- Decrease in compressive and flexural strength, modulus of elasticity, and bond strength with an increase in the quantity of recycled plastics. - Granule-type exhibited better compressive strength followed by flake- and fiber-type. - Flake-type exhibited better flexural strength than granule- and fiber-type.
Polymer concrete with recycled plastic—melting process	- Optimal range: 45–65% of the total mass - HDPE, LDPE investigated - Melting temperature: 100 °C - Dependent on curing method	- No cement or virgin polymers - Ductile behavior - Reduction in costs and impacts	- Possible impacts on workability and aggregates segregation (out of the optimal range) - Relatively low mechanical strength - High porosity
*Example* Plastic waste as the only binder to develop cemented construction material– PlasticWasteCrete (PWC) [118].	- Binding phase: two types of plastics (HDPE and LDPE) are blended, and melted at 250 °C. - PWC: binding phase mixed with mineral aggregates (sand and gravel). - No water is needed.	- Delay in thermal degradation. Inorganic materials (sand and gravel) as well as melting process contribute to the reinforcement and thermal stability of PWC. - Melted plastic binds granular materials with the presence of voids (the number of pores is less compared to conventional concrete due to the effect of water in Portland cement—SEM and MIP analysis). - Compressive strength: 10–15 MPa. Splitting tensile strength: 2–4 MPa. - Average density: 2.09–2.27 g/cm^3^. - 48 h immersion absorptions: 0.35% and 0.37%, very low compared to conventional cement concrete (4.58%).	- Melting process risks. - Long-term behavior and durability not fully understood.
Polymer concrete with recycled plastic—high pressure process	- Compaction process varying from 5 to 400 MPa investigated - Can be coupled with a thermal process	- Relatively good mechanical strength - Vibration-compacted concrete application - Low porosity	- Limitations in the practice - High impacts of the process
*Example* Compacted plastic concrete (CPC) alternative to traditional concrete [96].	- Recycled ordinary Portland cement concrete waste. - Recycled plastic waste PE. - Hot-pressing of recycled concrete powder (105–300 µm) with recycled plastic powder (65–150 °C and 10–50 MPa).	- Closed loop: recycling of concrete and a high-volume plastic waste. - Hot-pressing ensures a good fusion between concrete and plastic particles (SEM). - Superior bending strength than traditional concrete (5.5 MPa with 15% plastics—11 MPa with 25–75% plastic). - Less water is needed compared to traditional concrete. - Low porosity (SEM) confirms high durability and high frost-damage resistance.	- The effect of many factors like the type of plastic and the morphology of plastic are not fully understood. - Effects of recycling and hot-pressing of plastic.

## Data Availability

No new data were created or analyzed in this study.
